# Linderae Radix Attenuates Hyperlipidemia by Regulating Reverse Cholesterol Transport through the miR-33-PPARα/LXRα Axis

**DOI:** 10.2174/0118715303397929251030093605

**Published:** 2026-03-06

**Authors:** He Ye, Weizhi Lai, Shanshan Lei, Jiexian Zhang, Ke Li, Zhisen Wang, Xunjie Zheng, Xiaohu Yang, Zhaohuan Lou

**Affiliations:** 1 Department of Pharmacy, Zhejiang Hospital, Hangzhou, Zhejiang, 310013, China;; 2 School of Pharmaceutical Sciences, Zhejiang Chinese Medical University, Hangzhou, Zhejiang, 310053, China;; 3 Department of Medicine, Zhejiang Academy of Traditional Chinese Medicine, Hangzhou, Zhejiang, 310007, China;; 4 Department of Geriatrics, Taizhou First People’s Hospital, Taizhou, Zhengjiang, 318020, China

**Keywords:** Linderae Radix, hyperlipidemia, miR-33, PPARα, LXRα, reverse cholesterol transport

## Abstract

**Introduction:**

It has been reported that miR-33 could down-regulate the key protein expression of reverse cholesterol transport (RCT) to clear excess cholesterol. Our previous studies reported that Linderae Radix (Wuyao in Chinese, WY) has a strong anti-hyperlipidemia effect by regulating RCT, but whether the regulation of WY on RCT is related to miR-33 remains unclear.

**Methods:**

In *in vivo* research, mice were randomly divided into five groups. miR-33 agomir was administered to hyperlipidemic (HLP) mice to establish a miR-33 over-expression model. The anti-hyperlipidemic effect of WY in mice was evaluated by serum lipid profile, hematoxylin and eosin (H&E) staining, and Oil Red O staining. *In vitro*, the effect of WY on cholesteryl ester and uptake was measured in ox-LDL-induced RAW 264.7 cells. We established cell models with either overexpression or low expression of miR-33. The effect of WY on the expression of proteins related to reverse cholesterol transport (RCT) was evaluated using RT-qPCR, Western blotting, Wes automated protein expression analysis, and multiplex immunofluorescence assays.

**Results:**

WY could significantly reduce TC, TG, and LDL-C levels and improve lipid accumulation in the liver of both HLP mice and miR-33 over-expression HLP mice. Moreover, RCT-related proteins, such as ABCA1, ABCG1, PPARα, LXRα, and farnesoid X receptor (FXR), were also increased after WY treatment. The same trend was observed *in vitro*, that WY could alleviate lipid deposition in macrophages. Compared with the miR-33 mimic group, the expression of ABCA1, ABCG1, SR-BI, and LXRα was significantly increased in RAW264.7 cells incubated with WY. Compared with the miR-33 inhibitor group, WY markedly enhanced the expression levels of ABCA1, ABCG1, LXRα, and SR-BI in cells with low miR-33 expression.

**Discussion:**

Activation of PPARα/LXRα by Linderae Radix through miR-33 inhibition represents a mechanistically distinct approach from statins or fibrates, potentially benefiting statin-intolerant patients or serving as an adjunct therapy.

**Conclusion:**

These findings suggest that WY could attenuate hyperlipidemia by regulating RCT through the miR-33-PPARα/LXRα axis.

## 1 INTRODUCTION

Hyperlipidemia (HLP) refers to elevated levels of total cholesterol and/or triglycerides in the serum. Dyslipidemia, characterized by high cholesterol levels, is an important risk factor for atherosclerotic cardiovascular disease (ASCVD), such as coronary heart disease [1]. The effective prevention and management of HLP to reduce cardiovascular events have become an urgent priority. At present, chemical drugs for anti-hyperlipidemia, such as statins and fibrates, are the main drugs commonly prescribed. Chemical agents are effective in lowering lipid levels, but long-term use can lead to adverse reactions, such as liver and kidney injury and gastrointestinal reactions. Traditional Chinese medicine has the characteristics of multi-targeting and overall synergism and offers unique advantages in the treatment of hyperlipidemia. Further investigation into the mechanisms by which Chinese medicine effectively lowers blood lipids can facilitate the research and development of novel anti-hyperlipidemic Chinese medicines.

Studies have reported that cholesterol levels in the body are maintained relatively constant mainly through liver synthesis, intestinal absorption, and cholesterol clearance. It is known that the elimination of excess cholesterol in the body is mainly achieved through Reverse Cholesterol Transport (RCT), which is the central link of lipid metabolism and plays an important role in maintaining cholesterol homeostasis in the body, effectively preventing and treating hyperlipidemia and related atherosclerosis (AS) diseases [2]. High- density lipoprotein (HDL) is the primary executor of RCT [3]. Scavenger receptor class B type I (SR-BI) and Adenosine triphosphate binding cassette family A subfamily member 1 (ABCA1) [4] participate in the efflux of cholesterol in RCT. Peroxisome proliferator-activated receptor α (PPARα) and liver X receptor α (LXRα) are members of the nuclear receptor superfamily. Studies have confirmed that they could up-regulate the expression of ABCA1 and Adenosine triphosphate binding cassette transporter G1 (ABCG1) through the retinoid X receptor (RXR) \LXR pathway to promote cholesterol efflux [5, 6].

MicroRNA (miRNA) is a sort of single-stranded non- coding RNA composed of about 20-22 nucleotides. Most miRNAs can bind specifically to the 3' or 5' untranslated regions (UTRs) of mRNA, regulating gene expression after transcription by degrading mRNAs and suppressing their translation into protein [7]. A study [8] reported that miR-33 down-regulated the expression of RCT key enzymes, such as ABCA1 and ABCG1, at the post-transcriptional level, and another study [9] demonstrated that miR-33 could be efficiently delivered to macrophages *via* nanoparticles, where they could function to regulate ABCA1 expression and cholesterol efflux. Regulating the expression of key proteins during RCT can effectively modulate cholesterol transport *in vivo*, maintain cholesterol metabolism balance, and inhibit the onset and progression of HLP and related AS diseases.

Linderae Radix is the root of *Lindera aggregate (Sims)* Kosterm, which is called wuyao (WY) in Chinese, and is mainly produced in Tiantai, Zhejiang Province. It is one of the varieties cultivated for the new “Zhe Ba Wei” (Eight geo-authentic crude drugs in Zhejiang province), and commonly used for regulating qi in Chinese medicine practice. Recent studies have shown that WY can effectively protect the liver against lipid infiltration and improve lipid indices in HLP rats [10]. The extract of WY can significantly reduce the levels of serum TC and TG in the rat model with alcoholic liver injury [11]. Our previous studies found that the WY had definite lipid-lowering effects, reducing serum levels of TC, TG, and LDL-C in HLP model rats and up-regulating the expression of RCT-related proteins. Thus, in this research, we further explore whether WY regulation of RCT is associated with miR-33.

## 2 MATERIALS AND METHODS

### 2.1 Preparation of WY Extract and HPLC Analysis

The preparation of WY was as follows: The medicinal materials were provided by Zhejiang Hongshiliang Group Tiantaishan Wuyao Co., Ltd. (Batch No. 20211015). The original medicinal materials were soaked in 10 volumes of distilled water for 30 min and then boiled for 60 min using a reflux extraction device. The filtrate was collected, and 8 volumes of distilled water were added to the residue, which was then boiled for an additional 45 min. All the collected filtrate was combined and concentrated to an appropriate volume under vacuum at 55°C in a rotary evaporator. The liquid with a concentration of 200 mg/mL was stored at 4°C for later use.

The representative active ingredients (including Linderalactone, Linderane, and Lindenenol) in WY were identified by HPLC. The HPLC analysis was carried out using a Waters e2695 HPLC^®^ system. HPLC conditions were as follows: column, XB-C18 (4.6 × 250 mm, 5 μm, Welch Materials, Inc.); mobile phase, acetonitrile and H2O (56:44); column temperature, 30°C; injection volume, 10 µL; observed at 235nm. Reference standards of Linderalactone (B21561), Linderane (B21311), and Lindenenol (B21308) were purchased from Shanghai Yuanye Bio-Technology Co., Ltd, Shanghai, China.

### 2.2 Animals and Experimental Design

All specific pathogen-free (SPF) male C57BL/6J mice (weight 20±2 g, 8 weeks) were obtained from Hangzhou Qizhen Experimental Animal Technology Co., Ltd. The animal study was reviewed and approved by the Animal Ethics Committee of Hangzhou Qizhen Experimental Animal Technology Co., Ltd (No: 20220909). After adaptively fed for one week, all mice were randomly divided into five groups (n = 6 each group): normal control (NC) group, model control (MC) group, positive control group (Atorvastatin[Lipitor], 6.0 mg/kg), WY group (WY, 2.0 g/kg) and miR-33 ago+WY group (miR-33 agomir+WY, 2.0g/kg). The dosage was selected based on our previous research [10]. All mice received the drug *via* intragastric administration at a dose of 1 mL per 100 g of body weight once daily.

The NC group received a standard chow diet and water ad libitum, while the experimental cohort was fed a high-fat diet for 4 consecutive weeks to establish a hyperlipidemia mouse model. Mice in the miR-33 ago + WY group were intraperitoneally injected with 10 mg/kg miR-33 agomir (GenePharma, Shanghai, China), respectively. The other mice were intraperitoneally injected with normal saline once a week for 4 weeks.

A booster dose with the same amount was administered 2 days after the first injection in the first week. The drug intervention was given starting from the 5^th^ week for 4 consecutive weeks. In the end, the mice's orbital blood was collected and centrifuged, and the serum was obtained for testing. Liver tissues were dissected and divided into two aliquots: one was snap-frozen in liquid nitrogen for subsequent molecular analyses, while the other was fixed in 4% paraformaldehyde for 24 h at 4°C for histopathological examination.

### 2.3 Biochemical Biomarker Analysis

The serum levels of TG (DZ201), TC (DZ202), HDL-C (DZ203T), and LDL-C (DZ207) were measured using kits from the manufacturer (Ningbo Meikang Biotechnology Co., Ltd., China).

### 2.4 Histopathological Examination by Hematoxylin and Eosin (H&E)

The liver tissue was dehydrated with alcohol after being fixed in formalin and treated with xylene to become transparent. After that, it was embedded in paraffin, sectioned at 5 μm, stained with hematoxylin and eosin, and finally sealed onto the slide. The samples were analyzed using a B5-223IEP light microscope (Motic China Group Co., Ltd.).

### 2.5 Quantitative Real-time PCR (RT-qPCR)

Total RNA from mouse liver and RAW264.7 cells was extracted using the SPARK easily modified tissue and cellular RNA kit (AC0202, Sparkjade, China) according to the manufacturer’s instructions. Then, the cDNAs were obtained using Superscript II RT-PCR (Shandong Sparkjade Biotechnology Co., Ltd). The cDNA amplification was performed in triplicate using SYBR Green qPCR Mix (AH0104-B, Sparkjade, China) on an ABI 7500 real-time fluorescence quantitative PCR instrument (Applied Biosystems, USA). The β-actin, GAPDH, or U6 gene was used as a standardized control. The primer sequences are mentioned in Table **1****.**

### 2.6 Western Blot Analysis

Total protein from mouse liver tissues and RAW264.7 cells was extracted using RIPA lysis buffer. The protein concentration was determined by BCA (P0010, Beyotime Biotechnology, Shanghai, China). The same amount of total protein was taken from each sample, analyzed by 20% SDS- PAGE, and then transferred to a PVDF (IPVH00010, Millipore, USA) membrane. Blots were allowed to react with the specific primary antibodies: ABCA1 (96292, CST, USA), ABCG1 (ab52617, Abcam, Cambridge, UK), PPARα (ab126285, Abcam, Cambridge, UK), LXRα (14351-1-AP, Proteintech, Chicago, USA), FXR (ab155124, Abcam, Cambridge, UK), followed by crosslinking with the secondary antibodies. Protein expression was visualized with enhanced chemiluminescence reagent, and the band density was analyzed using the ImageJ software.

### 2.7 Cell Culture

RAW 264.7 macrophage was purchased from Cellverse Bioscience Technology Co., Ltd. (Shanghai, China) and was cultured in Dulbecco’s Modified Eagle’s medium (DMEM, 2022032902, Zhejiang Cienry Biotechnology Co., Ltd., containing 10% fetal bovine serum (Sangon, Shanghai, China)) at 37°C in a humidified atmosphere with 5% CO_2_. Overgrown cells were gently scraped off and collected into a centrifuge tube. The cell suspension was centrifuged at 1,200 rpm for 5 minutes, and after removing the supernatant, the pellet was resuspended in fresh complete medium. One-third of the suspension was then transferred into new culture flasks for continued expansion.

### 2.8 Cell Viability Determination

A cell suspension of RAW 264.7 cells was inoculated into 96-well plates and cultured in incubators. After the cells were fully attached to the wall, the cells were divided into 7 groups: the control group (DMEM medium), 1250 μg/mL, 625 μg/mL, 312 μg/mL, 156 μg/mL, 78 μg/mL, and 39 μg/mL of WY, and 6 compound pores were set up in each group. Following 24 or 48 h treatment periods, cellular morphology was assessed by microscopy, and viability was quantified using CCK-8 assays according to the manufacturer's protocol (CCK8, E606336-2000, Shanghai Sangon Biological Engineering Co., LTD, China). Then, 10 μL of CCK-8 reagent was added to each well and incubated for 1 h. OD value of each well was measured at 450 nm using an enzyme-linked microplate reader. The cell proliferation rate was calculated using the following formula:

Cell proliferation rate = (experimental group - control group)/control group × 100%

### 2.9 Determination of Cholesteryl Ester Content of RAW264.7 Cells

The concentration for cell treatment was selected based on the gradient concentrations used in previous experiments. RAW264.7 cells were inoculated in 24-well plates. They were randomly divided into the control group (normal cultured), ox-LDL group (100 μg/mL ox-LDL), ox-LDL+WYH group (100 μg/mL ox-LDL+156 μg/mL WY), ox-LDL+WYM group (100 μg/mL ox-LDL+78 μg/mL WY), and ox-LDL +WYL group (100 μg/mL ox-LDL+39 μg/mL WY), with each group plated in triplicate wells. After 24 h of intervention, the cells were collected and centrifuged. Anhydrous ethanol was added for the ultrasonic extraction of TC. The contents of TC and free cholesterol (FC) were determined according to the instructions of the lipid assay kit. To determine the content of cholesteryl ester, the following formula was used:

Cholesteryl ester (CE) content =TC-FC

### 2.10 Lipid Deposition Assay in RAW264.7 Cells by Oil Red O Staining

Oil red O staining was performed 24 hours after drug intervention. Specific steps were as follows: RAW264.7 cells were washed three times with PBS, fixed in 4% paraformaldehyde, stained with oil red O solution, decolorized with 60% isopropyl alcohol, and then stained with hematoxylin. Finally, the percentage of oil red in cells was calculated using ImageJ 6.0.

### 2.11 Lipid Uptake Assay in RAW264.7 Cells

RAW264.7 cells were inoculated in 24-well plates and randomly divided into the control group, ox-LDL group (100 μg/mL ox-LDL), ox-LDL+WYH group (100 μg/mL ox-LDL+156 μg/mL WY), ox-LDL+WYM group (100 μg/mL ox-LDL+78 μg/mL WY), and ox-LDL +WYL group (100 μg/mL ox-LDL+39 μg/mL WY), with each group plated in triplicate wells. After 24 h of drug intervention, fluorescence imaging was performed by a high-content cell imaging system, and fluorescence intensity was calculated by integrating optical density.

### 2.12 Preparation of Cell Models with High/Low Expression of miR-33

With reference to the literature [12-14], the transfection procedure was performed as follows: Transfection Solution A was prepared by mixing 660 μL of serum-free DMEM with 42 μL of Transmante and allowing the mixture to stand for 5 minutes at room temperature. Transfection Solution B was prepared by mixing 695 μL of serum-free DMEM with 7 μL of miR-33 (inhibitor or mimic, Lot: lss304, Suzhou Biosyntech Co., Ltd, China) and allowing it to stand for 5 minutes.

Solution B was then added to Solution A, mixed thoroughly, and allowed to stand at room temperature for 20 minutes to form Solution C. The medium in the 6-well plate was discarded, and 1.8 mL of DMEM containing 10% FBS was added to each well. Subsequently, 200 μL of Solution C was added to each well. The cells were incubated for 24 hours, after which the culture medium was removed.

RNA was then extracted from the cells, and the expression levels were analyzed by RT-PCR.

### 2.13 Wes Automated Protein Expression Analysis

Protein expression of ABCA1, ABCG1, and SR-BI was detected using the Wes automated protein expression analysis system in RAW264.7 cells. After 24 h of intervention, cells were collected with a scraper and centrifuged at 1250 rpm for 5 min. Then, 100 μL of RIPA lysate containing protease and phosphatase inhibitors was added, and ultrasonic extraction was carried out in the ice box for 1 min and suspended for 5s every 30s. The cell lysis solution was placed in a refrigerator at 4°C and mixed every 10 minutes to ensure complete lysis. After 40 min, the supernatant was collected by centrifugation at 12000 rpm for 10 min, and the cell concentration was detected by BCA. The corresponding protein expression was detected using the Wes automated protein expression analysis system.

### 2.14 Multiple-immunofluorescence Staining

Protein expression levels of ABCA1, ABCG1, and LXRα in RAW264.7 macrophages were quantitatively analyzed using tyramide signal amplification (TSA) multiplex immunofluorescence staining (Haoke Biotechnology, HKI000-5A), with DAPI counterstaining for nuclear localization. In short, the cells were placed in a 6-well plate and cultured in a cell incubator at 37°C. When the cells reached 80% confluence, they were divided into groups and processed according to the procedures for Oil Red O staining and the preparation of cell models with high or low expression of miR-33, as described previously. Cells were fixed by adding 4% formaldehyde in PBS for 20 min and washed three times with PBS. The cells were then permeabilized with 0.5% Triton X-100 for 20 min. ABCA1, ABCG1, and LXRα antibodies (1:200) were added and incubated at 4°C overnight, washed with PBS 3 times, and the corresponding secondary antibodies were added and incubated at room temperature for 1h. DAB was used for color development, and hematoxylin was used for nucleation. The excess stain was washed away, the sections were allowed to dry, and photographs were taken.

### 2.15 Statistical Analysis

SPSS 15.0 software was used for statistical analysis. Quantitative data are presented as mean ± standard deviation (mean ± SD). Intergroup comparisons were analyzed using Student's t-test for normally distributed data. One-way analysis of variance (ANOVA) was used to compare the multiple groups. *P* < 0.05 was considered statistically significant.

## 3 RESULTS

### 3.1 Identification of Major Components in Linderae Radix (WY)

**Fig. (1):**
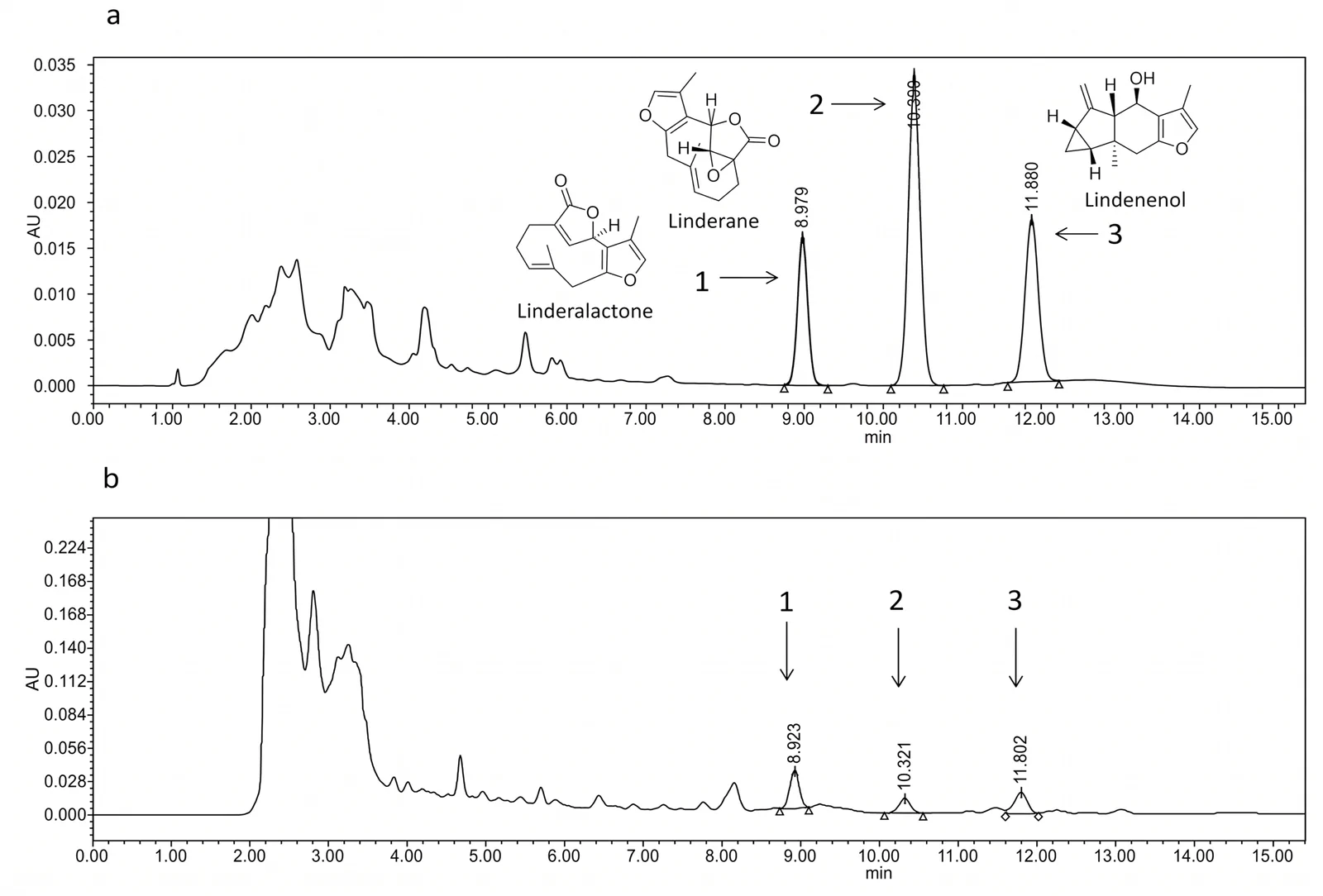
The HPLC chromatograms of the standard substances (**a**) and WY (**b**).

The primary active components of WY were identified by HPLC analysis and comparison with standard substances. These constituents mainly included linderalactone, linderane, and lindenenol (Fig. **1**).

### 3.2 WY Extract Reduced the Blood Lipid Levels and Ameliorated Liver Injury in HLP Model Mice

**Fig. (2):**
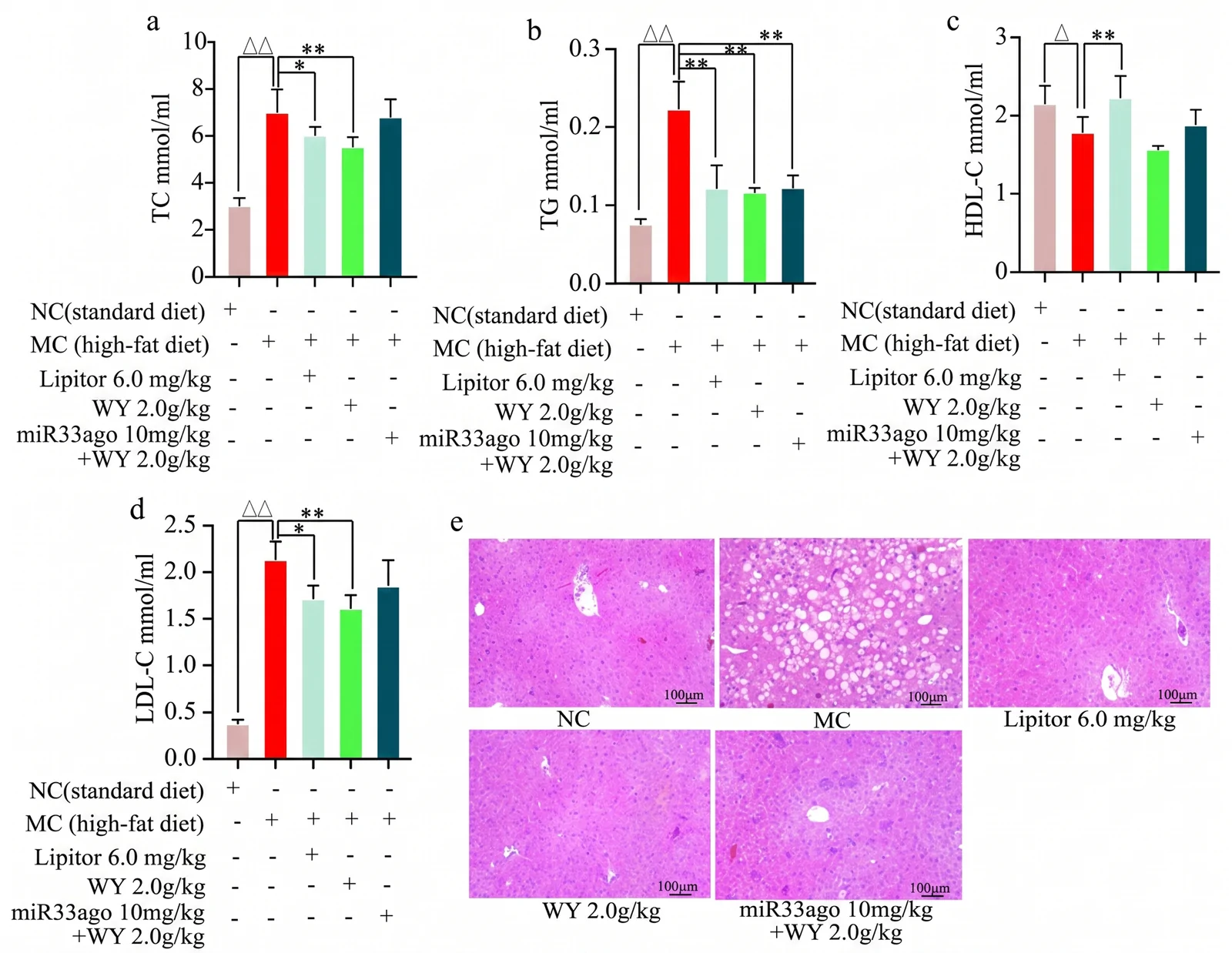
The effect of WY on blood lipid and liver histopathology in HLP mice. (**a**) TC; (**b**) TG; (**c**) HLD-C; (**d**) LDL-C; (**e**) Liver histopathology (H&E, 100×). Compared to the NC group, ^∆^*p* < 0.05, ^∆∆^*p* < 0.01; compared to the MC group, *p < 0.05, **p < 0.01.

Compared with the NC group, the serum levels of TC and TG in the MC group were significantly increased (*p* < 0.01, Figs. **2a** and **b**). The level of HDL-C was significantly decreased in the HLP model mice (*p* < 0.05, Fig. **2c**). In contrast, the serum level of LDL-C in the MC group was markedly increased (*p* < 0.01, Fig. **2d**). Compared with the MC group, WY could significantly reduce the serum levels of TC, TG, and LDL-C in HLP model mice (*p* < 0.01, Figs. **2a**, **b**, and **d**). While the effect of WY on TC and LDL-C disappeared in the miR-33 ago combined HLP mice.

Liver histopathology showed that, compared with the NC, the mice in the model control group exhibited numerous small fat droplets, inflammatory foci, and nuclear shrinkage. Compared with MC, lipid droplet accumulation and inflammatory infiltration improved in the WY group (Fig. **2e**). The inflammatory lesions in the liver tissue of miR33 ago + WY group mice were further increased, and fat droplets were reduced to a certain extent.

### 3.3 WY Regulated the miR-33-PPARα/LXRα Axis of the Liver in HLP Model Mice

**Fig. (3):**
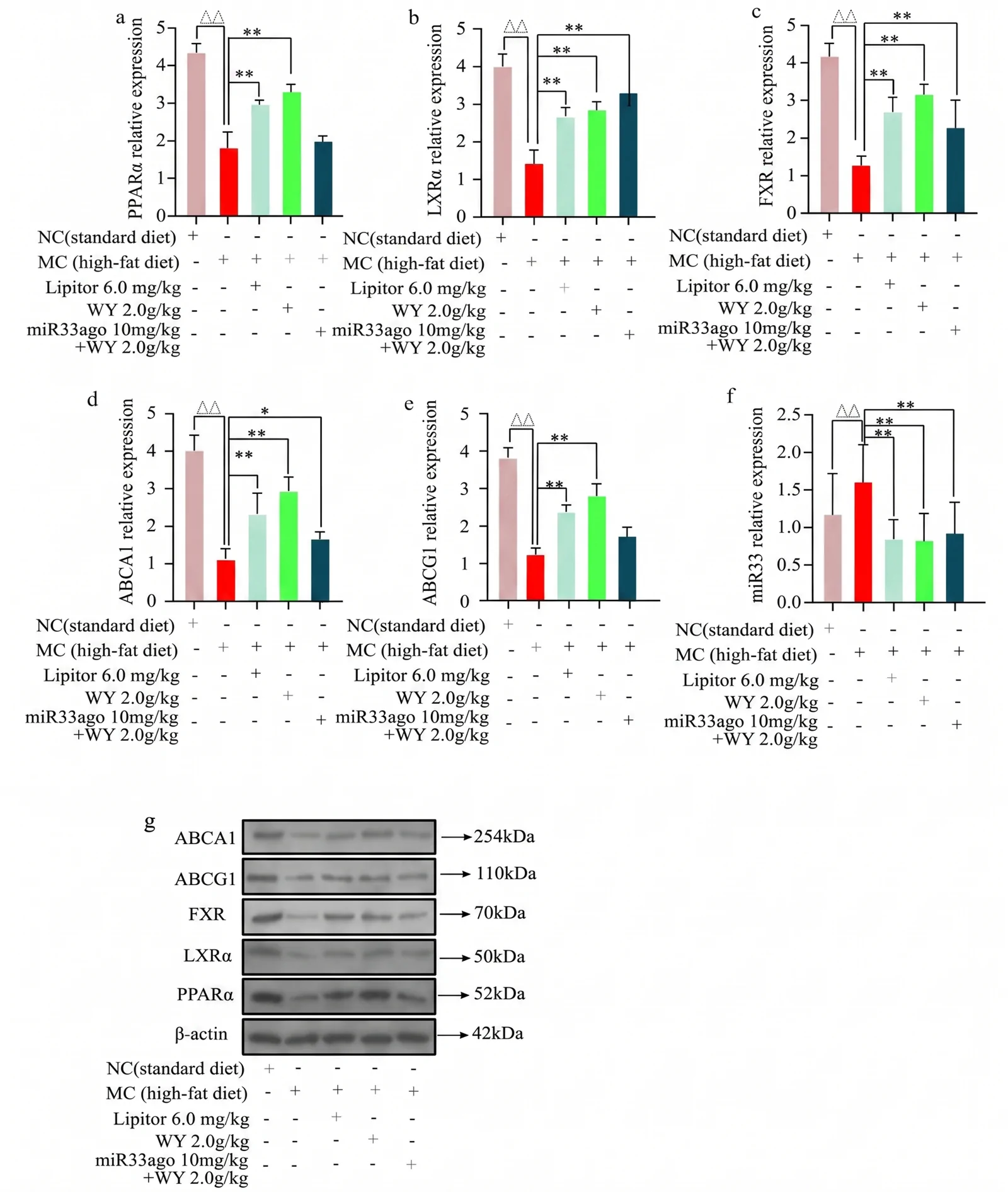
Effect of WY on protein expression was relative to the miR-33-PPARα/LXRα axis in HLP model mice. (**a**) PPARα relative expression. (**b**) LXRα relative expression. (**c**) FXR relative expression. (**d**) ABCA1 relative expression. (**e**) ABCG1 relative expression. (**f**) miR-33 relative expression. (**g**) Representative images showing the expression of ABCA1, ABCG1, FXR, LXRα, and PPARα proteins. Compared to the NC group, ^∆∆^*p* < 0.01; compared to the MC group, *p < 0.05, **p < 0.01.

The Western blot results showed that compared with the NC group, the protein expression of ABCA1, ABCG1, FXR, LXRα, and PPARα in the MC group was significantly down-regulated (*p* < 0.01, Figs. **3a**-**e**). Compared with the MC group, WY administration could significantly increase the protein expression of ABCA1, ABCG1, FXR, LXRα, and PPARα (*p* < 0.01, Figs. **3a**-**e**). However, the increase rate of ABCA1, ABCG1, FXR, and PPARα in the miR33 ago + WY group was lower than that in the WY-alone group. The qPCR result showed that miR-33 was significantly up-regulated in the MC group, and WY administration could reverse this effect (*p* < 0.01, Fig. **3f**). Representative images showing the expression of ABCA1, ABCG1, FXR, LXRα, and PPARα proteins are presented in Fig. (**3g**). These results indicated that WY regulated the PPARα/LXRα axis by inhibiting miR-33.

### 3.4 Effect of WY on RAW 264.7 Cells Proliferation

**Fig. (4):**
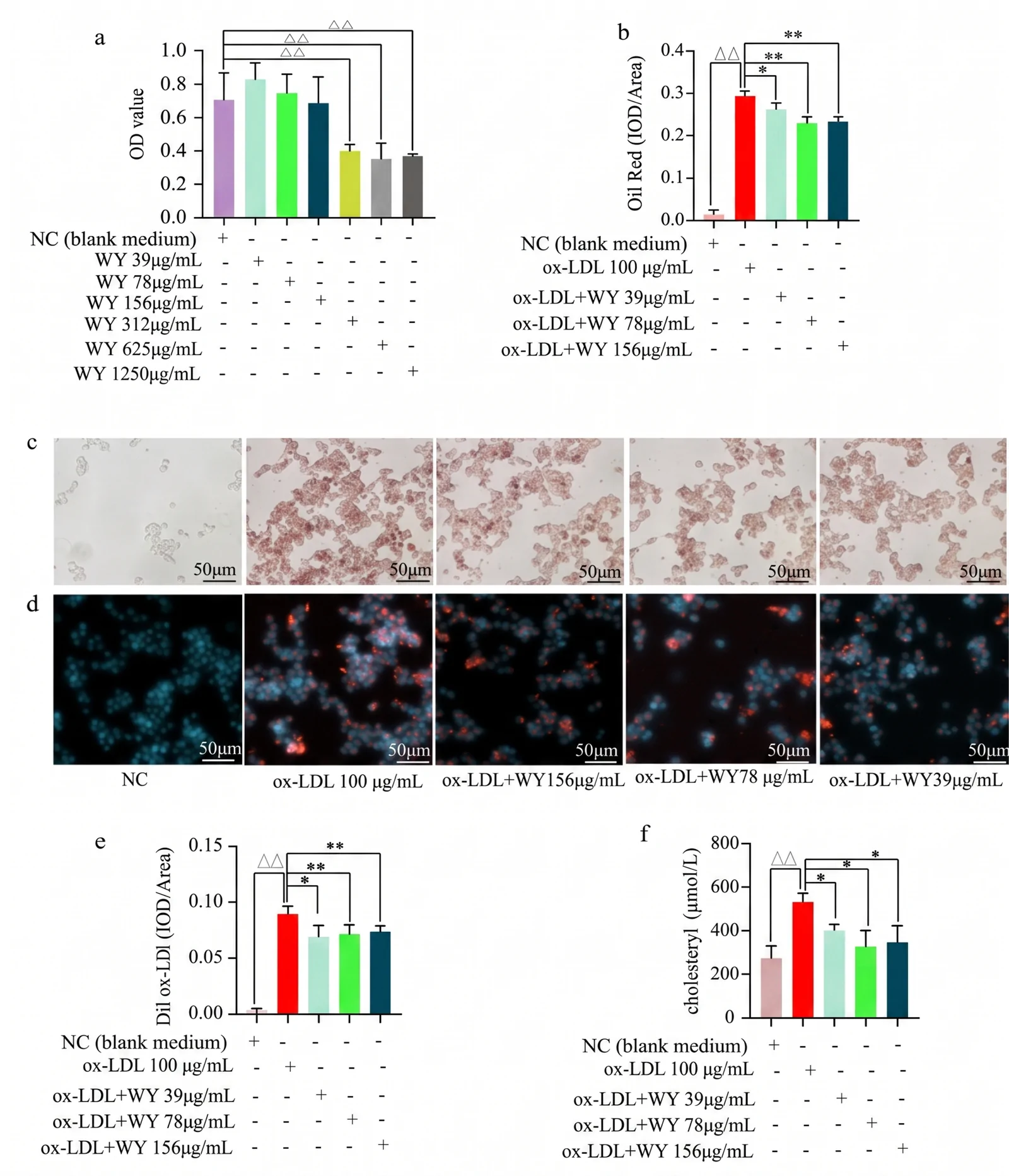
Effect of WY on the proliferative activity and RCT in RAW 264.7 cells. (**a**) Effect of WY on the proliferative activity of RAW 264.7 cells. (**b**) Oil Red (IOD/Area). (**c**) Effect of WY on lipid deposition in ox-LDL-induced RAW 264.7 cells. (**d**) Fluorescent cell imaging of WY on the uptake of ox-LDL in the RAW 264.7 cells using High Content Analysis. (**e**) Dil-ox-LDL (IOD/Area). (**f**) Effect of WY on cholesteryl ester content in ox-LDL-induced foamy macrophages. Compared to the NC group, ^∆∆^*p* < 0.01; compared to the ox-LDL group, *p < 0.05, **p < 0.01.

In the *in vitro* experiment, compared with the control group, the dose of WY from 39 to 156 μg/mL had no significant effect on the proliferation of RAW 264.7 cells after 24 and 48 h administration (*p* > 0.05). However, WY doses ranging from 312 to 1250 μg/mL significantly inhibited RAW 264.7 cell proliferation (*p* < 0.01). The results indicated that WY had no significant effect on the proliferation of RAW 264.7 cells at doses below 156 μg/mL (Fig. **4a**). Based on this, doses of 156, 78, and 39 μg/mL were selected for subsequent experiments.

### 3.5 WY Regulated RCT in ox-LDL-induced RAW 264.7 Cells

Compared with the NC group, the ox-LDL induced a significant increase in lipid deposition (Figs. **4b** and **c**), lipid uptake (Figs. **4d** and **e**), and cholesteryl ester content (Fig. **4f**) (*p* < 0.05, 0.01). Compared with the ox-LDL group, WY significantly decreased the cholesteryl ester, lipid deposition, and lipid uptake in a dose-dependent relationship (*p* < 0.05, 0.01). These results indicated that WY could regulate cholesterol RCT in the RAW 264.7 cells.

### 3.6 Effect of WY on the Expression of the PPARα/LXRα Axis in miR-33 Overexpression RAW 264.7 Cells

**Fig. (5):**
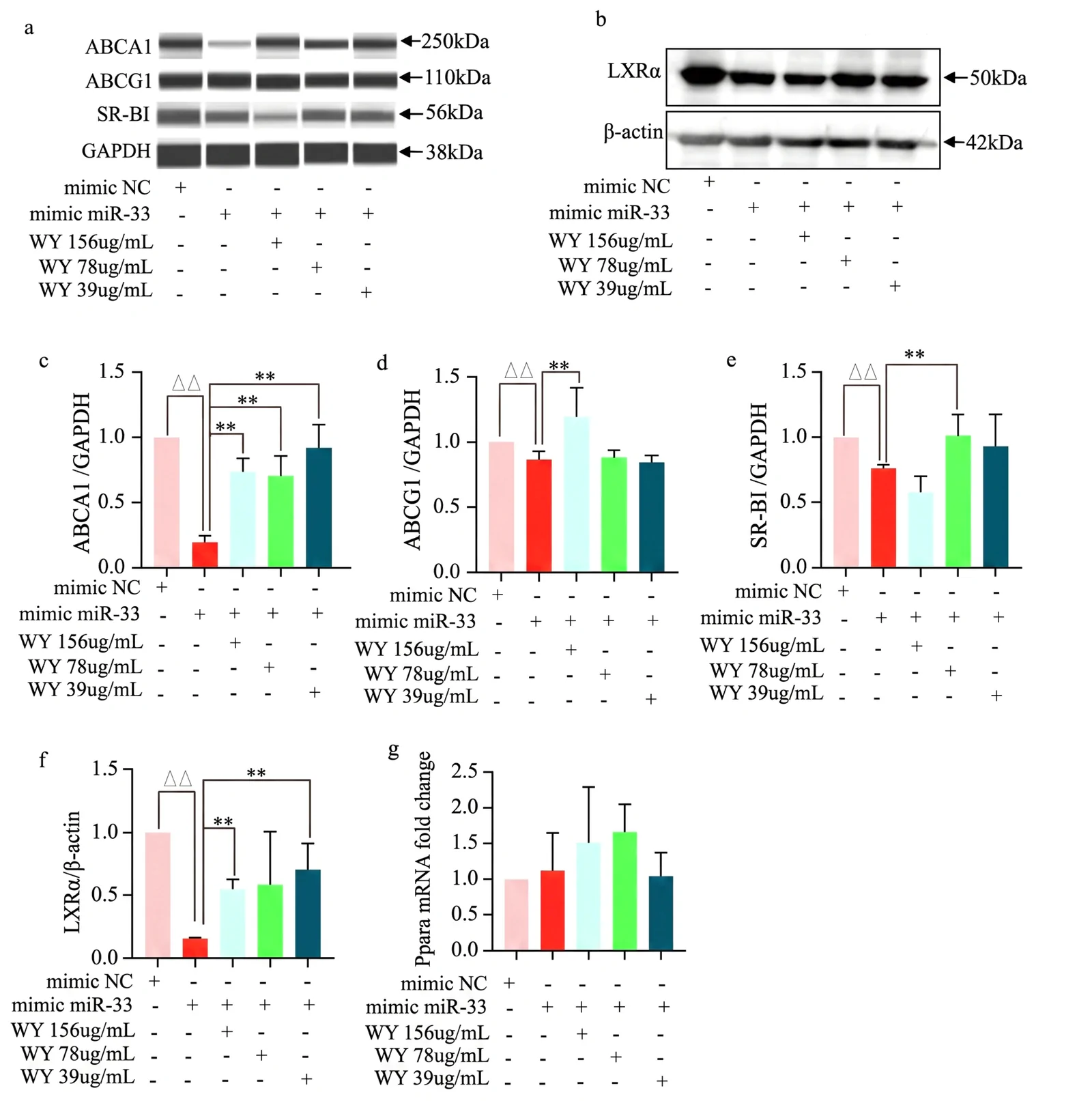
Effect of WY on the expression of the PPARα/LXRα axis in miR-33 over-expression cells. (**a**) The respective blots of ABCA1, ABCG1, and SR-BI proteins. (**b**) The respective blot of LXRα protein. (**c**) ABCA1/GAPDH. (**d**) ABCG1/GAPDH. (**e**) SR-BI/GAPDH. (**f**) LXRα/β-actin. (**g**) PPARα mRNA fold change. Compared to the mimic NC group, ^∆∆^*p* < 0.01; compared to the mimic miR-33 group, **p < 0.01.

To investigate whether WY regulates RCT *via* miR-33, we examined the effect of WY on the expression of the PPARα/LXRα axis in RAW 264.7 cells overexpressing miR-33. Compared with the mimic NC group, the protein expression results showed that the expression of ABCA1, ABCG1, SR-BI, and LXRα was significantly down-regulated in the miR-33 over-expression group (*p* < 0.01, Figs. **5a**-**f**). WYH (156 μg/mL) could significantly increase the expression levels of ABCA1, ABCG1, and LXRα protein (*p* < 0.01, Figs. **5c**, **d** and **f**). WYM (78 μg/mL) could up-regulate the expression of ABCA1 and SR-BI (*p* < 0.01, Figs. **5c** and **e**); moreover, WYL (39 μg/mL) could significantly increase the expression levels of ABCA1 and LXRα protein (*p* < 0.01, Figs. **5c** and **f**). Compared with the mimic NC group, PPARα gene expression in the miR-33 overexpression group showed no significant difference (Fig. **5g**). These results indicated that miR-33 could regulate RCT relative protein or gene expression, and WY reversed these changes by down-regulating miR-33 expression.

### 3.7 Effects of WY on the Protein Expression of LXRα/ABCA1/ABCG1/SR-BI in miR-33 Low-expression Cells

**Fig. (6):**
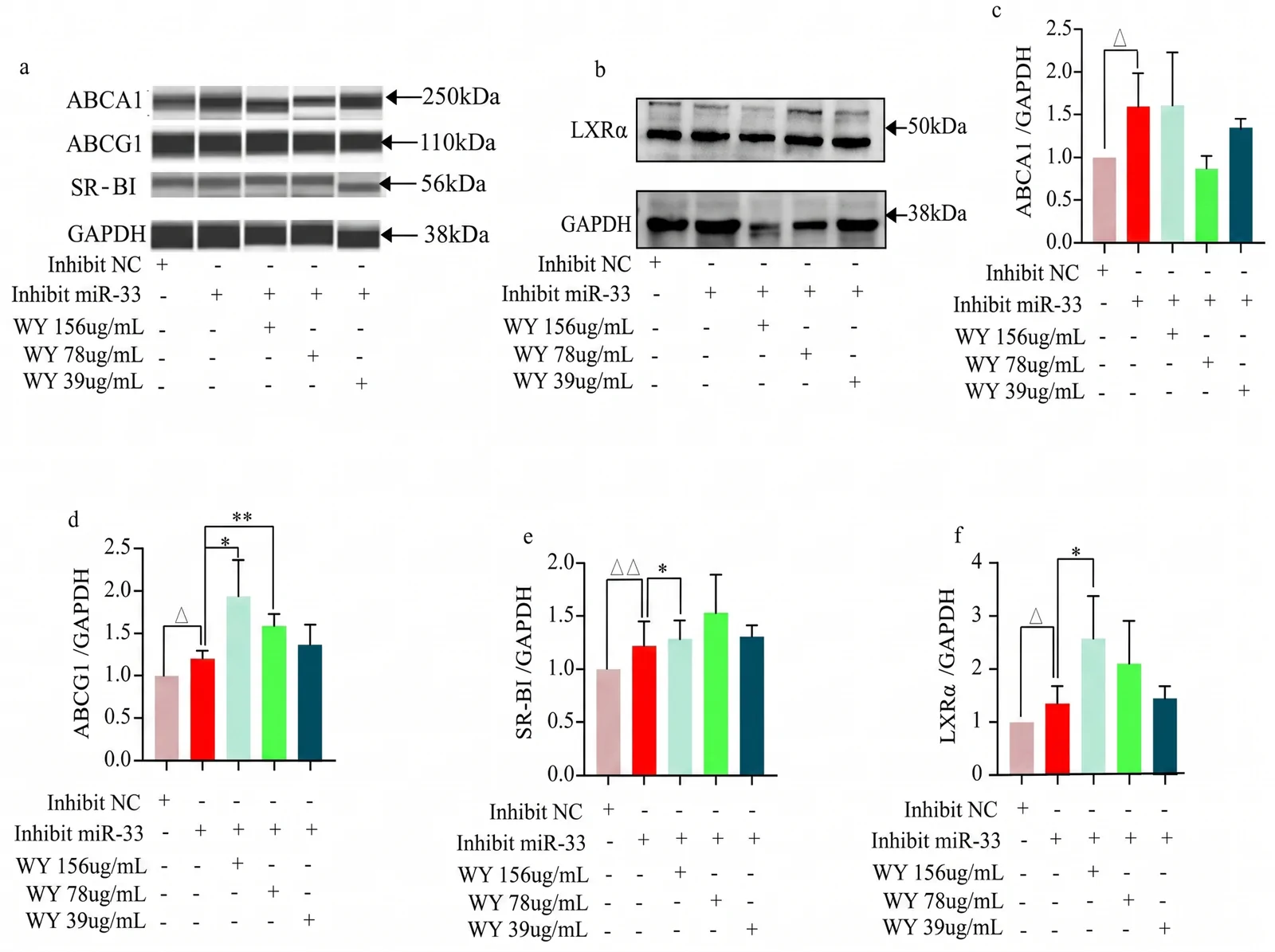
Effect of WY on the protein expression of LXRα/ABCA1/SR-BI in miR-33 low-expression cells. (**a**) The respective blots of ABCA1, ABCG1, and SR-BI. (**b**) The respective blot of LXRα protein. (**c**) ABCA1/GAPDH. (**d**) ABCG1/GAPDH. (**e**) SR-BI/GAPDH. (**f**) LXRα/GAPDH. Compared to the inhibited NC group, ^∆^*p* < 0.05, ^∆∆^*p* < 0.01; compared to the inhibited miR-33 group, *p < 0.05, **p < 0.01.

Compared with the inhibited NC group, the protein expression of ABCA1, ABCG1, SR-BI, and LXRα in the miR-33 low-expression cells was significantly up-regulated (*p* < 0.05, 0.01, Figs. **6a**-**f**). Compared with the miR-33 inhibited group, WYH (156 μg/mL) and WYM (78 μg/mL) could significantly increase the expression of ABCG1 (*p* < 0.05, 0.01, Figs. **6a** and **d**). WYH (156 μg/mL) could increase the expression levels of SR-BI and LXRα remarkably (*p* < 0.05, Figs. **6a**, **b**, **e** and **f**). However, WY had no significant effect on ABCA1 protein expression (Fig. **6c**). This confirmed that WY could regulate RCT *via* miR-33.

**Fig. (7):**
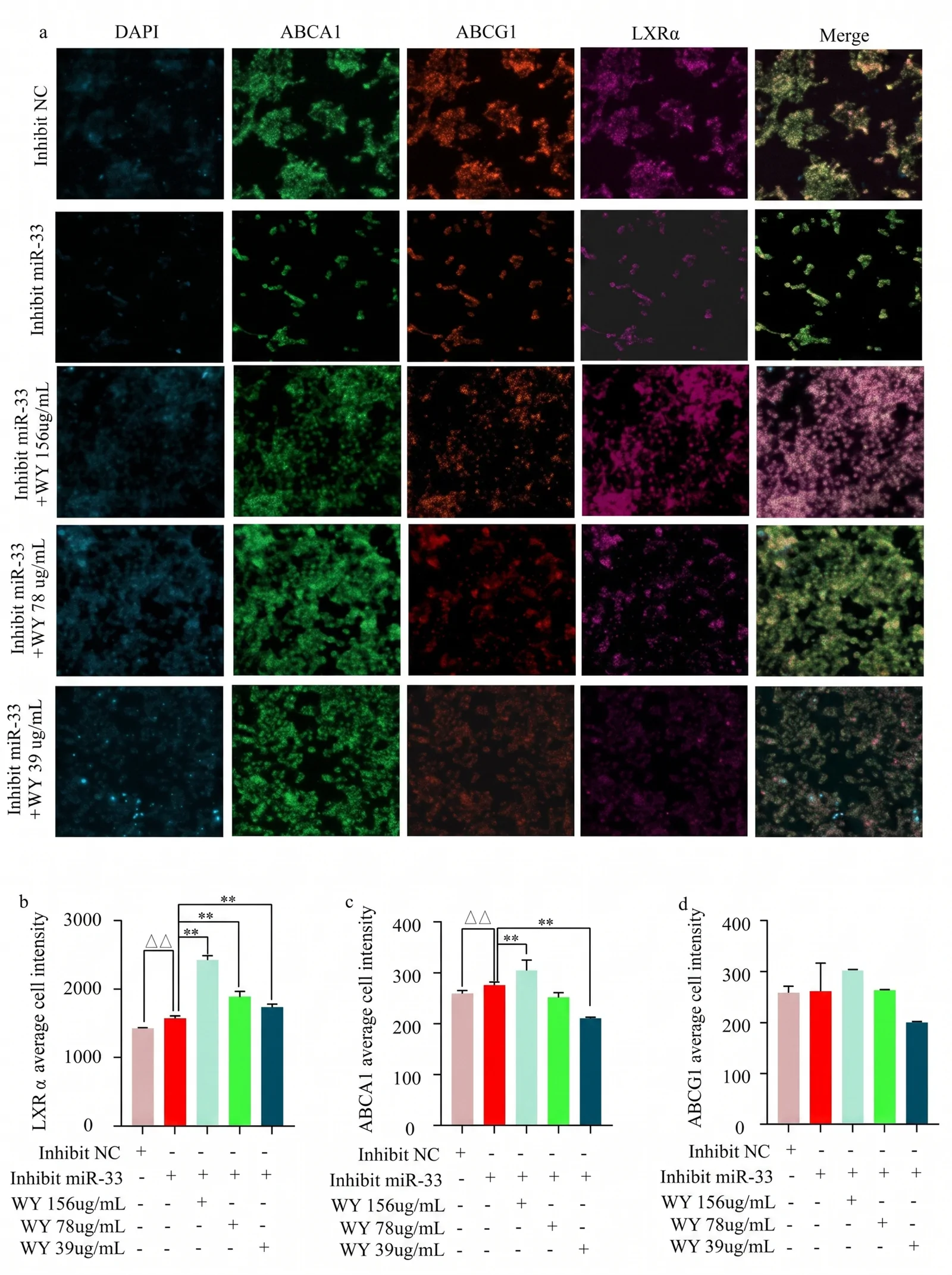
(**a**) Fluorescent imaging of ABCA1, ABCG1, and LXRα expression in ox-LDL–induced RAW 264.7 cells treated with WY. (**b**) LXRα average cell intensity. (**c**) ABCA1 average cell intensity. (**d**) ABCG1 average cell intensity. Compared to the inhibited NC group, ^∆∆^*p* < 0.01; compared to the inhibited miR-33 group, **p < 0.01.

Compared with the inhibited NC group, the protein expression of ABCA1 and LXRα in RAW 264.7 cells with low miR-33 expression was significantly up-regulated (*p* < 0.01, Figs. **7a**-**c**). Compared with the inhibited miR-33 group, WYH (156 μg/mL) and WYL (39 μg/mL) could significantly increase the protein expression levels of ABCA1 and LXRα (*p* < 0.01, Figs. **7a**-**c**). In addition, WYM (78 μg/mL) could significantly increase the expression level of LXRα protein (*p* < 0.01, Figs. **7a** and **b**). However, the protein expression of ABCG1 did not change significantly in any group of RAW 264.7 cells with low miR-33 expression (Fig. **7d**).

## 4 DISCUSSION

In this study, we established a hyperlipidemia animal model and administered WY extract as the intervention. The serum levels of TC, TG, and LDL-C in hyperlipidemia model mice were significantly increased, while HDL-C levels were significantly decreased, indicating that the hyperlipidemia model was successfully established. Our previous [15-19] and current studies found that WY extract could regulate the levels of serum TC, TG, LDL-C, and HDL-C in HLP model mice, and play a role in lowering blood lipids. Compared with Lipitor (Atorvastatin, Western Medicine), WY extract also showed significant anti-hyperlipidemic effects, especially in reducing TC, TG, and LDL-C, but had no effect on increasing HDL-C. At present, the treatment of hyperlipidemia is mainly based on Western medicine, which often requires the combination of multiple drugs, such as statins (Atorvastatin, *etc*), combined with other lipid-lowering drugs. Although Western drugs have rapid anti-hyperlipidemia effects and positive efficacy, their long-term use often causes side effects. The most common side effect of statins is liver injury, and patients experience discomfort and pain. Traditional Chinese medicine (TCM) has the characteristics of “multi-component, multi-action, multi-target, and holistic regulation”, and its therapeutic effect is moderate, which has unique advantages in the treatment of hyperlipidemia. Therefore, in-depth research on the mechanism of lipid-lowering action of effective TCM will help to develop new TCM with unique anti-hyperlipidemia effects. Moreover, the lipid-lowering properties of WY extract make it a potential candidate drug for treating hyperlipidemia.

Oxidized low-density lipoprotein (ox-LDL), generated by the oxidative modification of elevated circulating LDL, accumulates in the vascular intima, where it induces endothelial dysfunction and initiates pro-inflammatory cascades *via* oxidative stress-mediated pathways. Macrophages, activated from monocytes, phagocytose ox-LDL through scavenger receptors to form foam cells, promoting the occurrence and development of AS [20]. The results of the present study showed that WY extract could inhibit cholesterol uptake by macrophages and improve lipid deposition in ox-LDL-induced foamy macrophages. It is suggested that WY extract has an antagonistic effect on hyperlipidemia and AS.

In addition, we conducted tests on SR-BI. As a specific HDL receptor, SR-BI plays a key role in HDL metabolism and RCT. The main role of hepatocyte SR-BI is to mediate the selective uptake of plasma HDL-C, remove excess cholesterol from peripheral tissues, convert it into bile, expel it from the body, and maintain the body's cholesterol balance. The regulation of its expression is directly related to the efficiency of RCT [21, 22]. In this study, we found that the protein expression of SR-BI in miR-33 over/low-expression RAW 264.7 cells was significantly increased after the intervention of WY, suggesting that the lipid-lowering effect of WY may be related to miR-33 expression.

Liver X receptors (LXRs) are a kind of nuclear receptor activated by oxidized sterols, which play an important role in RCT, glucose metabolism, and inflammation [23], and LXRα is one of its subtypes. Previous studies reported that LXRs promote the flow of cholesterol from the cell to the cell membrane and transport it to the cells by regulating target genes, such as ApoE, ABCA1, ABCG1, etc., and prevent the excessive accumulation of cholesterol in the vascular wall to prevent the occurrence and development of AS [24, 25]. Furthermore, LXRs functionally interact with the PPAR signaling pathway, serving as a critical regulatory node that integrates lipid metabolic processes. This coupling primarily modulates the transcriptional regulation of genes involved in fatty acid transport and β-oxidation, thereby maintaining systemic lipid homeostasis. Hepatic PPARα activity controls apolipoprotein expression and regulates ABCA1 in macrophages and the gut [26-28]. Another study [29] found that SAVEP could reduce the TC/TG index, repair nuclear damage, reduce lipid accumulation, and ultimately lower the apoptosis rate by up-regulating the expression of PPARα, LXRα, and ABCA1, key proteins in lipid metabolism. Furthermore, as members of the nuclear receptor superfamily, PPARα and LXRα have been demonstrated to cooperatively enhance ABCA1 and ABCG1 expression *via* the RXR/LXR heterodimerization pathway, thereby stimulating cellular cholesterol efflux mechanisms [30]. This study showed that WY effectively increased protein expression levels of LXRα and PPARα in HLP mice. These results suggested that WY might promote cholesterol efflux through the PPARα/LXRα axis.

To further investigate the lipid-lowering mechanism of WY, we specifically selected miR-33 agomir to upregulate the target gene and verified downstream genes and proteins associated with WY's lipid-lowering effect. In liver tissue from HLP mice, we found that WY significantly up-regulated the protein expression of ABCA1, ABCG1, LXRα, FXR, and PPARα in the miR33 ago + WY group. However, the increase rate of ABCA1, ABCG1, FXR, and PPARα in the miR33 ago + WY group was lower than that in the WY-administered alone group. These results indicated that WY regulated the PPARα/LXRα axis by inhibiting miR-33.

To verify whether WY exerts its anti-hyperlipidemic effects through the miR-33-PPARα/LXRα axis by modulating reverse cholesterol transport (RCT) *via* miR-33 inhibition, we investigated the regulatory effects of WY in RAW 264.7 macrophages with genetically manipulated miR-33 expression. The results indicated that WY significantly up-regulated the protein expression of ABCA1, ABCG1, LXRα, and SR-BI in miR-33 overexpression cells, and the gene expression of PPARα showed an increasing trend. Moreover, the results showed that WY significantly up-regulated the protein expression of ABCA1, ABCG1, LXRα, and SR-BI in miR-33-low expression RAW 264.7 cells. Based on the above results, we clarified that WY could upregulate the PPARα/LXRα signaling pathway-mediated RCT process by inhibiting miR-33.

The activation of PPARα/LXRα by Linderae Radix *via* miR-33 inhibition offers a mechanistically distinct approach compared to statins or fibrates. This multitargeted action enhances reverse cholesterol transport (RCT), addressing both cholesterol efflux and clearance. Clinically, this could benefit statin-intolerant patients or serve as an adjunct therapy. Future studies will aim to validate the significance of this axis in human primary hepatocytes and patient-derived organoids. This will thereby promote the clinical application of Linderae Radix and the development of its resources.

## 5 STUDY LIMITATIONS

This study has some limitations. The small sample size of the study may cause some bias. We only detected the representative protein and gene expression related to lipid metabolism; the roles of other related proteins and genes remain to be further elucidated. In addition, the regulation of the PPARα/LXRα axis by miR-33 needs further study.

Although this study has demonstrated good lipid-lowering effects of Linderae Radix in animal and cellular models, we have not yet obtained clinical research data due to limited resources. This limitation restricts its application for treating hyperlipidemia in patients. In future research, we may recruit relevant volunteers to conduct a study on its lipid-lowering efficacy.

## CONCLUSION

This study demonstrated that traditional Chinese medicine WY effectively reduced hyperlipidemia by improving the RCT process, including enhancing the expression of ABCA1, ABCG1, PPARα, LXRα, and FXR. The anti-hyperlipidemia mechanism is that WY attenuates hyperlipidemia by regulating RCT *via* the miR-33-PPARα/LXRα axis.

## Figures and Tables

**Table 1:** The primer sequences.

**Gene**	FORWARD (5’-3’)	REVERSE (3’-5’)
miR-33	GUGCAUUGUAGUUGCAUUGCA	CAAUGCAACUACAAUGCACUU
U6 ABCA1	TGCGGGTGTGTCGCTTCGGCAGC CATGGGAAGCTGTGTTGTGTG	CCAGTGCAGGGTCCGAGGT GGCCAGCATCAGAACTGCTC
ABCG1	CCTGCTGTACCTGGGGATTG	CCAGGGGAAAGGTCAGAACG
FXR	TCGTTCGGCGGAGATTTTCA	RCTGTGAGCAGAGCGTACTCC
LXRα	GGCAGTGAGAGCATCACCTT	TGATGGCAATGAGCAGAGCA
PPARα	TGAGGAAGCCGTTCTGTGAC	CACAATCCCCTCCTGCAACT
GAPDH	GGAGTCCACTGGTCTTCA	GGGAACTGGCAATTGGTGTGGG

## Data Availability

All data generated or analyzed during this study are included in this published article.

## LIST OF ABBREVIATIONS

ABCA1: ATP-binding Cassette Sub-family A Member 1
ABCG1: ATP-Binding Cassette Sub-family G Member 1
Apo-A1: Apolipoprotein A1
AS: Atherosclerosis
ASCVD: Atherosclerotic Cardiovascular Disease
CCK8: Cell Counting Kit-8
DMEM: Dulbecco’s Modified Eagle’s Medium
FXR: Farnesoid X Receptor
HDL-C: High-density Lipoprotein Cholesterol
H&E: Hematoxylin and Eosin
HLP: Hyperlipidemia
LDL-C: Low Density Lipoprotein Cholesterol
LXRα: Liver X Receptor α
ox-LDL: Oxidized-Low Density Lipoprotein
PBS: Phosphate-buffered saline
PPARα: Peroxisome Proliferator-activated Receptor α
RCT: Reverse Cholesterol Transport
RT-qPCR: Quantitative Real-time PCR
RXR: Retinoid X Receptor
SR-BI: Scavenger Receptor Class B type I
TC: Total Cholesterol
TG: Triglyceride
TSA: Tyramide Signal Amplification
